# 3-*tert*-Butyl 5-methyl (2*R*,4*S*,5*R*)-2-(4-methoxyphenyl)-4-(3-nitrophenyl)-1,3-oxazolidine-3,5-dicarboxylate

**DOI:** 10.1107/S160053681204192X

**Published:** 2012-10-20

**Authors:** Sara Montiel-Smith, Sylvain Bernès, Jesús Sandoval-Ramírez, Socorro Meza-Reyes, Joëlle Dubois

**Affiliations:** a Benemérita Universidad Autónoma de Puebla, Facultad de Ciencias Químicas, Ciudad Universitaria, Puebla, Pue. 72570, Mexico; b Universidad Autónoma de Nuevo León, UANL, Facultad de Ciencias Químicas, Av. Universidad S/N, Ciudad Universitaria, San Nicolás de los Garza, Nuevo León CP 66451, Mexico; c Institut de Chimie des Substances Naturelles, CNRS, Avenue de la Terrasse, 91190 Gif-sur-Yvette, France

## Abstract

The title mol­ecule, C_23_H_26_N_2_O_8_, was synthesized in three steps starting from *m*-nitro­cinnamic acid. The central oxazolidine ring adopts an almost perfect envelope conformation with the O atom as the flap [puckering parameter *ϕ* = 0.3 (6)°]. The dihedral angle formed by the benzene rings is 61.81 (9)°. In the crystal, mol­ecules are connected into double chains parallel to [010] by C—H⋯O hydrogen bonds. The absolute configuration was assigned from the synthetic procedure.

## Related literature

For the Sharpless asymmetric amino­hydroxy­lation, see: Rudolph *et al.* (1996 ▶). For the synthesis of the phenyl­isoserine precursor of the title mol­ecule, see: Montiel-Smith *et al.* (2002 ▶). For the stereocontrolled formation of the oxazolidine in the title mol­ecule, see: Denis *et al.* (1994 ▶). For the structure of a related chiral *N*-Boc-protected oxazolidine, see: Tinant *et al.* (1996 ▶). For puckering parameters, see: Cremer & Pople (1975 ▶).
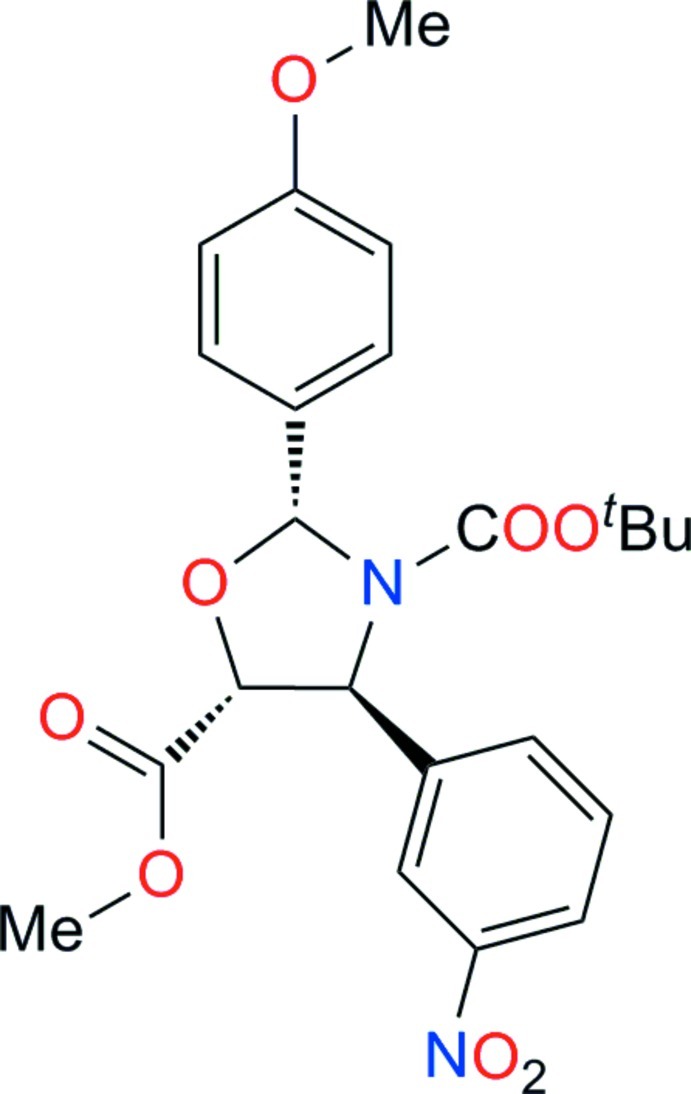


## Experimental

### 

#### Crystal data

C_23_H_26_N_2_O_8_
*M* *_r_* = 458.46
Monoclinic, 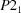

*a* = 10.383 (1) Å
*b* = 6.0303 (6) Å
*c* = 18.7366 (17) Å
β = 95.591 (4)°
*V* = 1167.57 (19) Å^3^
*Z* = 2
Mo *K*α radiation
μ = 0.10 mm^−1^
*T* = 298 K
0.60 × 0.16 × 0.16 mm

#### Data collection

Siemens P4 diffractometer
3169 measured reflections
2275 independent reflections
1628 reflections with *I* > 2σ(*I*)
*R*_int_ = 0.024
3 standard reflections every 97 reflections intensity decay: 0.5%

#### Refinement

*R*[*F* ^2^ > 2σ(*F* ^2^)] = 0.039
*wR*(*F* ^2^) = 0.095
*S* = 1.02
2275 reflections
304 parameters
1 restraint
H-atom parameters constrained
Δρ_max_ = 0.12 e Å^−3^
Δρ_min_ = −0.13 e Å^−3^

### 

Data collection: *XSCANS* (Siemens, 1996 ▶); cell refinement: *XSCANS*; data reduction: *XSCANS*; program(s) used to solve structure: *SHELXS97* (Sheldrick, 2008 ▶); program(s) used to refine structure: *SHELXL97* (Sheldrick, 2008 ▶); molecular graphics: *Mercury* (Macrae *et al.*, 2008 ▶); software used to prepare material for publication: *SHELXL97*.

## Supplementary Material

Crystal structure: contains datablock(s) I, global. DOI: 10.1107/S160053681204192X/rz5008sup1.cif

Structure factors: contains datablock(s) I. DOI: 10.1107/S160053681204192X/rz5008Isup2.hkl

Supplementary material file. DOI: 10.1107/S160053681204192X/rz5008Isup3.cml

Additional supplementary materials:  crystallographic information; 3D view; checkCIF report

## Figures and Tables

**Table 1:** Hydrogen-bond geometry (Å, °)

*D*—H⋯*A*	*D*—H	H⋯*A*	*D*⋯*A*	*D*—H⋯*A*
C2—H2*A*⋯O15^i^	0.98	2.50	3.400 (4)	153
C5—H5*A*⋯O28^ii^	0.98	2.59	3.387 (4)	138
C26—H26*A*⋯O1^iii^	0.93	2.59	3.252 (4)	128

Symmetry codes: (i) 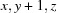; (ii) 
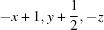
; (iii) 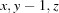.

**Fig. 1.**
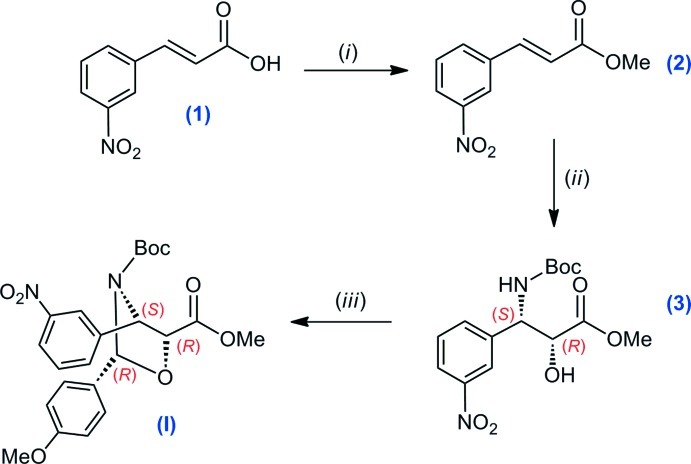
The 3-steps synthesis of the title molecule, (I). i) SOCl2, MeOH, reflux; ii) (DHQ)2PHAL, n-PrOH/tBuOCONHCl; K2OsO2(OH)4, 0 °C; iii) p-MeOC6H4CH(OMe)2, PPTS, toluene, 80 °C.

**Fig. 2.**
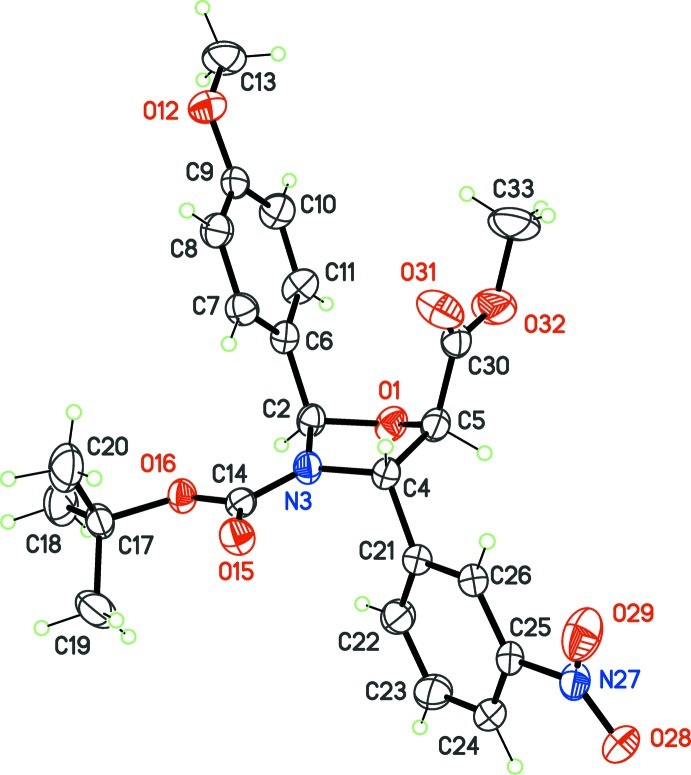
ORTEP-like view of the title molecule, showing 30% displacement ellipsoids for non-H atoms.

**Fig. 3.**
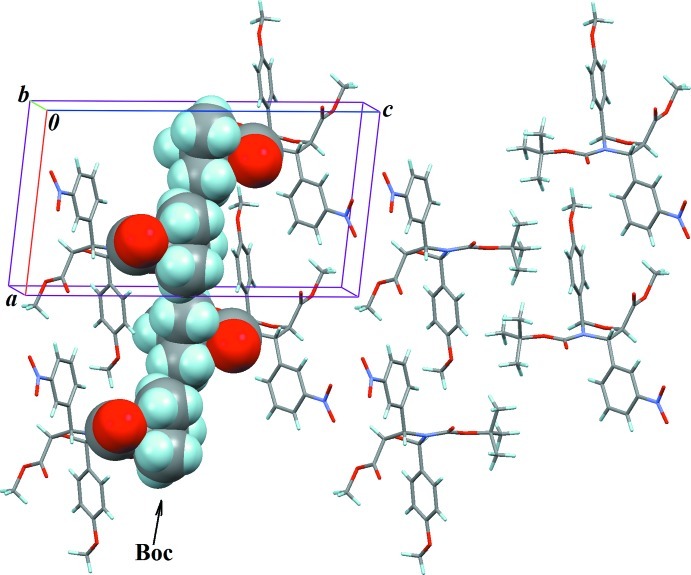
Part of the crystal structure of the title compound, viewed along the b axis. In four molecules, the Boc substituents are shown using a spacefill representation, in order to emphasize the stacking for these groups in the crystal.

## supplementary crystallographic information

### Comment

The title compound is related to a project about new synthetic routes to obtain isoserines (*α*-hydroxy-*β*-amino acids). The stereocontrol of the synthesis is a key point, since chiral isoserines are found in bioactive substances, as in the side chain of the emblematic anti-cancer agent Paclitaxel, initially marketed under the brand name Taxol. We focused our efforts toward the synthesis of (2*R*,3*S*)-*N*-Boc-*β*-phenylisoserines (Montiel-Smith *et al.*, 2002). Starting from commercially available *m*-nitrocinnamic acid, which was esterified in a first step, we probed various conditions for an asymmetric aminohydroxylation (Rudolph *et al.*, 1996), and the best results were obtained by using *tert*-butyl-*N*-chlorocarbamate as the nitrogen source, (DHQ)_2_PHAL (hydroquinine 1,4-phthalazinediyl diether) as chiral ligand, and K_2_OsO_2_(OH)_4_ as catalyst. The desired phenylisoserine was eventually obtained with 81% *ee* (see compound 2c in Montiel-Smith *et al.*, 2002). The title compound resulted from the protection of the amine and hydroxyl groups, *via* the formation of an oxazolidine (Fig. 1).

The molecular structure (Fig. 2) allowed to check for the configuration of chiral atoms C2 and C3 in the precursor isoserine 3, confirming that the chiral inductor (DHQ)_2_PHAL affords the (2*R*,3*S*) isomer predominantly, as expected. The deduced configuration of the third stereocenter in the oxazolidine I, 2*R*, also agrees with literature data for related reactions (Denis *et al.*, 1994). The substituents in the oxazolidine skeleton are arranged in such a way that steric hindrance is avoided. The oxazolidine exhibits a conformation very close to the ideal envelope conformation on O1, the puckering parameters (Cremer & Pople, 1975) being *φ* = 0.3 (6)° and *q*_2_ = 0.331 (3) Å. The ring conformation is related to the substituents distribution. For instance, the X-ray structure for another *N*-Boc protected oxazolidine with a different absolute configuration, (2*R*,4*R*,5*S*), showed a twisted oxazolidine ring (Tinant *et al.*, 1996).

The crystal structure (Fig. 3) is dominated by the stacking of bulky Boc groups, which are oriented along [100], with the molecules linked into double chains parallel to [010] by C—H···O hydrogen bonds (Table 1).

### Experimental

The synthesis starting from commercially available *m*-nitrocinnamic acid 1 is depicted in Fig. 1. The two steps preparation of the phenylisoserine 3 has been published (Montiel-Smith *et al.*, 2002; see compound 2c therein). The enantiospecific aminohydroxylation reaction was carried out using *^t^*BuOCONHCl as nitrogen source, reagent previously prepared *in situ* by reacting *^t^*BuOCONH_2_ and *^t^*BuOCl in a NaOH solution. The last step to afford the title compound is a protection of the amine and hydroxyl groups of 3, *via* the formation of an oxazolidine, carried out by reacting 3 with 1-(dimethoxymethyl)-4-methoxybenzene in presence of pyridinium *p*-toluenesulfonate, in refluxing toluene. The isolated compound I was crystallized from AcOEt/heptane.

### Refinement

The assignment of the absolute configuration of the three chiral centers was based on the stereospecificity of the synthetic pathway. The second synthetic step (see Fig. 1) allows to fix the stereochemistry for C4 and C5 centers. The last chiral center on C2, formed in the third step, is assigned as 2*R* relatively to the (4*S*,5*R*) stereoisomer. The reaction afforded a single stereoisomer. The formation of the chiral center 2*R* in I is in agreement with reports for related compounds (Denis *et al.*, 1994). Measured Friedel pairs are not suitable for checking this assignation, and were merged (425 pairs). All H atoms were placed in idealized positions, with C—H bond lengths fixed to 0.98 (methine CH), 0.96 (methyl CH_3_, rigid groups free to rotate about the C—C bonds) or 0.93 Å (aromatic CH). Isotropic displacement parameters for H atoms were calculated as *U*_iso_(H) = *xU*_eq_(carrier C), where *x* = 1.5 (methyl groups) or 1.2 (aromatic and methine CH).

### Crystal data

**Table:**

C_23_H_26_N_2_O_8_	*F*(000) = 484
*M**_r_* = 458.46	*D*_x_ = 1.304 Mg m^−^^3^
Monoclinic, *P*2_1_	Melting point = 388–391 K
Hall symbol: P 2yb	Mo *K*α radiation, λ = 0.71073 Å
*a* = 10.383 (1) Å	Cell parameters from 58 reflections
*b* = 6.0303 (6) Å	θ = 3.9–11.9°
*c* = 18.7366 (17) Å	µ = 0.10 mm^−^^1^
β = 95.591 (4)°	*T* = 298 K
*V* = 1167.57 (19) Å^3^	Block, colourless
*Z* = 2	0.60 × 0.16 × 0.16 mm

### Data collection

**Table:**

Siemens P4 diffractometer	*R*_int_ = 0.024
Radiation source: fine-focus sealed tube	θ_max_ = 25.0°, θ_min_ = 2.0°
Graphite monochromator	*h* = −12→1
ω scans	*k* = −7→1
3169 measured reflections	*l* = −22→22
2275 independent reflections	3 standard reflections every 97 reflections
1628 reflections with *I* > 2σ(*I*)	intensity decay: 0.5%

### Refinement

**Table:**

Refinement on *F*^2^	Secondary atom site location: difference Fourier map
Least-squares matrix: full	Hydrogen site location: inferred from neighbouring sites
*R*[*F*^2^ > 2σ(*F*^2^)] = 0.039	H-atom parameters constrained
*wR*(*F*^2^) = 0.095	*w* = 1/[σ^2^(*F*_o_^2^) + (0.0467*P*)^2^] where *P* = (*F*_o_^2^ + 2*F*_c_^2^)/3
*S* = 1.02	(Δ/σ)_max_ < 0.001
2275 reflections	Δρ_max_ = 0.12 e Å^−^^3^
304 parameters	Δρ_min_ = −0.13 e Å^−^^3^
1 restraint	Extinction correction: *SHELXL97*, Fc^*^=kFc[1+0.001xFc^2^λ^3^/sin(2θ)]^-1/4^
0 constraints	Extinction coefficient: 0.017 (3)
Primary atom site location: structure-invariant direct methods	

### Fractional atomic coordinates and isotropic or equivalent isotropic displacement parameters (Å^2^)

**Table:**

	*x*	*y*	*z*	*U*_iso_*/*U*_eq_	
O1	0.7821 (2)	0.5989 (4)	0.18897 (11)	0.0582 (7)	
C2	0.8184 (3)	0.5427 (6)	0.26275 (16)	0.0467 (8)	
H2A	0.7769	0.6446	0.2942	0.056*	
N3	0.7630 (3)	0.3207 (5)	0.26723 (13)	0.0474 (7)	
C4	0.7412 (3)	0.2150 (6)	0.19661 (14)	0.0457 (8)	
H4A	0.7970	0.0844	0.1947	0.055*	
C5	0.7866 (3)	0.3983 (7)	0.14856 (17)	0.0543 (9)	
H5A	0.7241	0.4104	0.1061	0.065*	
C6	0.9643 (3)	0.5490 (5)	0.28030 (15)	0.0440 (8)	
C7	1.0343 (3)	0.3772 (6)	0.31379 (17)	0.0508 (9)	
H7A	0.9914	0.2492	0.3258	0.061*	
C8	1.1675 (3)	0.3915 (7)	0.32989 (17)	0.0549 (9)	
H8A	1.2129	0.2732	0.3520	0.066*	
C9	1.2322 (3)	0.5807 (7)	0.31311 (16)	0.0519 (9)	
C10	1.1655 (4)	0.7536 (7)	0.27930 (19)	0.0628 (10)	
H10A	1.2090	0.8805	0.2668	0.075*	
C11	1.0321 (4)	0.7370 (6)	0.26391 (19)	0.0603 (10)	
H11A	0.9870	0.8558	0.2419	0.072*	
O12	1.3643 (2)	0.5782 (5)	0.33188 (12)	0.0708 (8)	
C13	1.4373 (4)	0.7685 (8)	0.3159 (2)	0.0840 (14)	
H13A	1.5271	0.7437	0.3311	0.126*	
H13B	1.4071	0.8949	0.3406	0.126*	
H13C	1.4269	0.7953	0.2651	0.126*	
C14	0.7368 (3)	0.2149 (6)	0.32801 (17)	0.0446 (8)	
O15	0.7056 (2)	0.0214 (4)	0.32967 (12)	0.0564 (6)	
O16	0.7505 (2)	0.3553 (4)	0.38389 (10)	0.0502 (6)	
C17	0.7426 (3)	0.2749 (6)	0.45810 (16)	0.0556 (10)	
C18	0.7608 (4)	0.4849 (8)	0.5014 (2)	0.0852 (14)	
H18A	0.6974	0.5923	0.4834	0.128*	
H18B	0.8460	0.5428	0.4975	0.128*	
H18C	0.7506	0.4536	0.5507	0.128*	
C19	0.6121 (4)	0.1736 (10)	0.4654 (2)	0.0896 (16)	
H19A	0.5457	0.2681	0.4428	0.134*	
H19B	0.6000	0.1575	0.5152	0.134*	
H19C	0.6072	0.0307	0.4427	0.134*	
C20	0.8534 (4)	0.1162 (10)	0.4763 (2)	0.0901 (14)	
H20A	0.8410	−0.0143	0.4471	0.135*	
H20B	0.8566	0.0757	0.5260	0.135*	
H20C	0.9333	0.1864	0.4674	0.135*	
C21	0.6011 (3)	0.1515 (6)	0.17565 (15)	0.0439 (8)	
C22	0.4983 (3)	0.2716 (7)	0.19742 (18)	0.0613 (10)	
H22A	0.5140	0.3881	0.2296	0.074*	
C23	0.3719 (3)	0.2199 (8)	0.17162 (19)	0.0719 (12)	
H23A	0.3036	0.3021	0.1866	0.086*	
C24	0.3470 (3)	0.0468 (8)	0.12382 (18)	0.0637 (11)	
H24A	0.2627	0.0117	0.1060	0.076*	
C25	0.4499 (3)	−0.0711 (7)	0.10355 (15)	0.0494 (9)	
C26	0.5756 (3)	−0.0233 (6)	0.12914 (15)	0.0465 (9)	
H26A	0.6432	−0.1089	0.1150	0.056*	
N27	0.4263 (3)	−0.2579 (6)	0.05319 (14)	0.0626 (9)	
O28	0.3196 (3)	−0.2710 (6)	0.01928 (13)	0.0901 (11)	
O29	0.5151 (3)	−0.3873 (6)	0.04674 (14)	0.0893 (9)	
C30	0.9204 (4)	0.3588 (8)	0.12370 (18)	0.0605 (11)	
O31	0.9865 (3)	0.1999 (6)	0.13651 (16)	0.0899 (10)	
O32	0.9520 (3)	0.5284 (7)	0.08509 (16)	0.1015 (11)	
C33	1.0810 (4)	0.5279 (14)	0.0607 (3)	0.137 (3)	
H33A	1.0921	0.6582	0.0326	0.206*	
H33B	1.0913	0.3983	0.0321	0.206*	
H33C	1.1446	0.5267	0.1015	0.206*	

### Atomic displacement parameters (Å^2^)

**Table:**

	*U*^11^	*U*^22^	*U*^33^	*U*^12^	*U*^13^	*U*^23^
O1	0.0616 (14)	0.0538 (16)	0.0564 (13)	−0.0020 (14)	−0.0088 (11)	0.0128 (14)
C2	0.0548 (19)	0.042 (2)	0.0421 (17)	−0.0031 (18)	−0.0010 (14)	0.0022 (17)
N3	0.0571 (17)	0.0470 (17)	0.0376 (14)	−0.0109 (15)	0.0019 (12)	0.0019 (14)
C4	0.0458 (18)	0.052 (2)	0.0385 (16)	−0.0010 (18)	−0.0007 (13)	−0.0027 (17)
C5	0.054 (2)	0.063 (3)	0.0437 (17)	−0.008 (2)	−0.0041 (15)	0.005 (2)
C6	0.0543 (18)	0.036 (2)	0.0411 (16)	−0.0041 (18)	0.0005 (15)	0.0003 (16)
C7	0.053 (2)	0.046 (2)	0.0542 (19)	−0.0040 (19)	0.0073 (16)	0.0064 (18)
C8	0.057 (2)	0.054 (2)	0.054 (2)	0.004 (2)	0.0032 (16)	0.0095 (19)
C9	0.051 (2)	0.066 (3)	0.0385 (16)	−0.007 (2)	0.0045 (15)	−0.0062 (19)
C10	0.067 (2)	0.051 (2)	0.069 (2)	−0.016 (2)	0.0007 (18)	0.006 (2)
C11	0.063 (2)	0.042 (2)	0.073 (2)	−0.004 (2)	−0.0075 (18)	0.005 (2)
O12	0.0519 (15)	0.085 (2)	0.0746 (15)	−0.0143 (17)	0.0006 (12)	0.0006 (17)
C13	0.062 (2)	0.091 (4)	0.100 (3)	−0.033 (3)	0.014 (2)	−0.018 (3)
C14	0.0409 (18)	0.047 (2)	0.045 (2)	−0.0020 (19)	0.0015 (14)	−0.0019 (19)
O15	0.0693 (15)	0.0461 (16)	0.0547 (14)	−0.0124 (14)	0.0099 (11)	−0.0018 (12)
O16	0.0613 (14)	0.0495 (14)	0.0393 (11)	−0.0080 (13)	0.0026 (10)	−0.0022 (11)
C17	0.065 (2)	0.064 (3)	0.0383 (17)	−0.011 (2)	0.0088 (16)	0.0024 (19)
C18	0.108 (3)	0.092 (4)	0.055 (2)	−0.026 (3)	0.006 (2)	−0.017 (2)
C19	0.091 (3)	0.113 (4)	0.071 (2)	−0.035 (3)	0.038 (2)	−0.016 (3)
C20	0.104 (3)	0.103 (4)	0.061 (2)	0.011 (3)	−0.006 (2)	0.022 (3)
C21	0.0427 (18)	0.049 (2)	0.0398 (15)	−0.0022 (17)	0.0029 (14)	0.0001 (17)
C22	0.055 (2)	0.070 (3)	0.059 (2)	0.005 (2)	0.0047 (17)	−0.012 (2)
C23	0.049 (2)	0.096 (3)	0.072 (2)	0.014 (2)	0.0076 (18)	−0.011 (3)
C24	0.045 (2)	0.096 (3)	0.0491 (18)	−0.007 (2)	0.0001 (16)	−0.004 (2)
C25	0.052 (2)	0.063 (2)	0.0326 (15)	−0.0074 (19)	0.0009 (14)	−0.0010 (17)
C26	0.047 (2)	0.054 (2)	0.0382 (16)	−0.0030 (17)	0.0044 (14)	0.0000 (17)
N27	0.068 (2)	0.080 (3)	0.0386 (15)	−0.023 (2)	−0.0005 (15)	−0.0020 (18)
O28	0.0673 (17)	0.139 (3)	0.0621 (15)	−0.040 (2)	−0.0047 (13)	−0.0214 (19)
O29	0.105 (2)	0.087 (2)	0.0720 (17)	0.006 (2)	−0.0127 (16)	−0.0255 (19)
C30	0.061 (3)	0.077 (3)	0.0432 (19)	−0.016 (3)	0.0004 (17)	0.007 (2)
O31	0.079 (2)	0.099 (3)	0.098 (2)	0.005 (2)	0.0398 (16)	0.007 (2)
O32	0.0703 (18)	0.138 (3)	0.0973 (19)	−0.023 (2)	0.0104 (15)	0.049 (2)
C33	0.066 (3)	0.223 (8)	0.127 (4)	−0.030 (4)	0.031 (3)	0.062 (5)

### Geometric parameters (Å, º)

**Table:**

O1—C5	1.430 (4)	C17—C18	1.506 (5)
O1—C2	1.437 (4)	C17—C20	1.510 (6)
C2—N3	1.462 (5)	C18—H18A	0.9600
C2—C6	1.519 (4)	C18—H18B	0.9600
C2—H2A	0.9800	C18—H18C	0.9600
N3—C14	1.356 (4)	C19—H19A	0.9600
N3—C4	1.466 (4)	C19—H19B	0.9600
C4—C21	1.518 (4)	C19—H19C	0.9600
C4—C5	1.529 (5)	C20—H20A	0.9600
C4—H4A	0.9800	C20—H20B	0.9600
C5—C30	1.526 (5)	C20—H20C	0.9600
C5—H5A	0.9800	C21—C26	1.377 (4)
C6—C7	1.381 (4)	C21—C22	1.384 (5)
C6—C11	1.385 (5)	C22—C23	1.390 (5)
C7—C8	1.389 (5)	C22—H22A	0.9300
C7—H7A	0.9300	C23—C24	1.383 (6)
C8—C9	1.376 (5)	C23—H23A	0.9300
C8—H8A	0.9300	C24—C25	1.368 (5)
C9—C10	1.372 (5)	C24—H24A	0.9300
C9—O12	1.383 (4)	C25—C26	1.376 (4)
C10—C11	1.391 (5)	C25—N27	1.474 (5)
C10—H10A	0.9300	C26—H26A	0.9300
C11—H11A	0.9300	N27—O29	1.223 (4)
O12—C13	1.423 (5)	N27—O28	1.224 (4)
C13—H13A	0.9600	C30—O31	1.189 (5)
C13—H13B	0.9600	C30—O32	1.313 (5)
C13—H13C	0.9600	O32—C33	1.456 (5)
C14—O15	1.212 (4)	C33—H33A	0.9600
C14—O16	1.343 (4)	C33—H33B	0.9600
O16—C17	1.483 (4)	C33—H33C	0.9600
C17—C19	1.504 (5)		
			
C5—O1—C2	106.9 (2)	C19—C17—C18	111.1 (3)
O1—C2—N3	101.7 (2)	O16—C17—C20	107.9 (3)
O1—C2—C6	111.4 (2)	C19—C17—C20	113.3 (4)
N3—C2—C6	113.6 (3)	C18—C17—C20	111.0 (3)
O1—C2—H2A	110.0	C17—C18—H18A	109.5
N3—C2—H2A	110.0	C17—C18—H18B	109.5
C6—C2—H2A	110.0	H18A—C18—H18B	109.5
C14—N3—C2	126.3 (3)	C17—C18—H18C	109.5
C14—N3—C4	121.8 (3)	H18A—C18—H18C	109.5
C2—N3—C4	111.9 (3)	H18B—C18—H18C	109.5
N3—C4—C21	113.8 (2)	C17—C19—H19A	109.5
N3—C4—C5	100.8 (3)	C17—C19—H19B	109.5
C21—C4—C5	111.9 (2)	H19A—C19—H19B	109.5
N3—C4—H4A	110.0	C17—C19—H19C	109.5
C21—C4—H4A	110.0	H19A—C19—H19C	109.5
C5—C4—H4A	110.0	H19B—C19—H19C	109.5
O1—C5—C30	111.8 (3)	C17—C20—H20A	109.5
O1—C5—C4	105.8 (2)	C17—C20—H20B	109.5
C30—C5—C4	114.1 (3)	H20A—C20—H20B	109.5
O1—C5—H5A	108.3	C17—C20—H20C	109.5
C30—C5—H5A	108.3	H20A—C20—H20C	109.5
C4—C5—H5A	108.3	H20B—C20—H20C	109.5
C7—C6—C11	117.3 (3)	C26—C21—C22	118.7 (3)
C7—C6—C2	123.3 (3)	C26—C21—C4	118.5 (3)
C11—C6—C2	119.4 (3)	C22—C21—C4	122.6 (3)
C6—C7—C8	121.4 (3)	C21—C22—C23	120.6 (4)
C6—C7—H7A	119.3	C21—C22—H22A	119.7
C8—C7—H7A	119.3	C23—C22—H22A	119.7
C9—C8—C7	120.0 (3)	C24—C23—C22	120.4 (4)
C9—C8—H8A	120.0	C24—C23—H23A	119.8
C7—C8—H8A	120.0	C22—C23—H23A	119.8
C10—C9—C8	120.0 (3)	C25—C24—C23	118.0 (3)
C10—C9—O12	124.7 (4)	C25—C24—H24A	121.0
C8—C9—O12	115.3 (4)	C23—C24—H24A	121.0
C9—C10—C11	119.2 (4)	C24—C25—C26	122.3 (3)
C9—C10—H10A	120.4	C24—C25—N27	119.2 (3)
C11—C10—H10A	120.4	C26—C25—N27	118.5 (3)
C6—C11—C10	122.0 (4)	C25—C26—C21	119.9 (3)
C6—C11—H11A	119.0	C25—C26—H26A	120.0
C10—C11—H11A	119.0	C21—C26—H26A	120.0
C9—O12—C13	118.2 (3)	O29—N27—O28	124.0 (4)
O12—C13—H13A	109.5	O29—N27—C25	118.1 (3)
O12—C13—H13B	109.5	O28—N27—C25	117.9 (4)
H13A—C13—H13B	109.5	O31—C30—O32	124.7 (4)
O12—C13—H13C	109.5	O31—C30—C5	126.1 (4)
H13A—C13—H13C	109.5	O32—C30—C5	109.3 (4)
H13B—C13—H13C	109.5	C30—O32—C33	117.2 (5)
O15—C14—O16	126.5 (3)	O32—C33—H33A	109.5
O15—C14—N3	123.4 (3)	O32—C33—H33B	109.5
O16—C14—N3	110.0 (3)	H33A—C33—H33B	109.5
C14—O16—C17	120.9 (3)	O32—C33—H33C	109.5
O16—C17—C19	110.5 (3)	H33A—C33—H33C	109.5
O16—C17—C18	102.4 (3)	H33B—C33—H33C	109.5
			
C5—O1—C2—N3	−34.8 (3)	C4—N3—C14—O15	7.2 (5)
C5—O1—C2—C6	86.6 (3)	C2—N3—C14—O16	9.3 (4)
O1—C2—N3—C14	−160.3 (3)	C4—N3—C14—O16	−172.8 (3)
C6—C2—N3—C14	80.0 (4)	O15—C14—O16—C17	7.2 (5)
O1—C2—N3—C4	21.7 (3)	N3—C14—O16—C17	−172.8 (2)
C6—C2—N3—C4	−98.1 (3)	C14—O16—C17—C19	−60.6 (4)
C14—N3—C4—C21	60.8 (4)	C14—O16—C17—C18	−179.0 (3)
C2—N3—C4—C21	−121.0 (3)	C14—O16—C17—C20	63.8 (4)
C14—N3—C4—C5	−179.3 (3)	N3—C4—C21—C26	−152.8 (3)
C2—N3—C4—C5	−1.1 (3)	C5—C4—C21—C26	93.8 (4)
C2—O1—C5—C30	−89.2 (3)	N3—C4—C21—C22	31.9 (4)
C2—O1—C5—C4	35.6 (3)	C5—C4—C21—C22	−81.6 (4)
N3—C4—C5—O1	−20.3 (3)	C26—C21—C22—C23	−1.4 (5)
C21—C4—C5—O1	100.9 (3)	C4—C21—C22—C23	174.0 (3)
N3—C4—C5—C30	103.0 (3)	C21—C22—C23—C24	0.1 (6)
C21—C4—C5—C30	−135.8 (3)	C22—C23—C24—C25	0.5 (6)
O1—C2—C6—C7	−129.1 (3)	C23—C24—C25—C26	0.1 (5)
N3—C2—C6—C7	−15.0 (4)	C23—C24—C25—N27	179.4 (3)
O1—C2—C6—C11	52.5 (4)	C24—C25—C26—C21	−1.4 (5)
N3—C2—C6—C11	166.7 (3)	N27—C25—C26—C21	179.3 (3)
C11—C6—C7—C8	−0.5 (5)	C22—C21—C26—C25	2.0 (4)
C2—C6—C7—C8	−178.9 (3)	C4—C21—C26—C25	−173.6 (3)
C6—C7—C8—C9	0.7 (5)	C24—C25—N27—O29	−165.8 (3)
C7—C8—C9—C10	−1.2 (5)	C26—C25—N27—O29	13.5 (5)
C7—C8—C9—O12	179.6 (3)	C24—C25—N27—O28	15.7 (5)
C8—C9—C10—C11	1.5 (5)	C26—C25—N27—O28	−164.9 (3)
O12—C9—C10—C11	−179.3 (3)	O1—C5—C30—O31	122.5 (4)
C7—C6—C11—C10	0.9 (5)	C4—C5—C30—O31	2.5 (5)
C2—C6—C11—C10	179.3 (3)	O1—C5—C30—O32	−57.8 (4)
C9—C10—C11—C6	−1.4 (5)	C4—C5—C30—O32	−177.8 (3)
C10—C9—O12—C13	0.6 (5)	O31—C30—O32—C33	−4.6 (6)
C8—C9—O12—C13	179.8 (3)	C5—C30—O32—C33	175.7 (4)
C2—N3—C14—O15	−170.7 (3)		

### Hydrogen-bond geometry (Å, º)

**Table:**

*D*—H···*A*	*D*—H	H···*A*	*D*···*A*	*D*—H···*A*
C2—H2*A*···O15^i^	0.98	2.50	3.400 (4)	153
C5—H5*A*···O28^ii^	0.98	2.59	3.387 (4)	138
C26—H26*A*···O1^iii^	0.93	2.59	3.252 (4)	128

Symmetry codes: (i) *x*, *y*+1, *z*; (ii) −*x*+1, *y*+1/2, −*z*; (iii) *x*, *y*−1, *z*.

## References

[bb1] Cremer, D. & Pople, J. A. (1975). *J. Am. Chem. Soc.* **97**, 1354–1358.

[bb2] Denis, J.-N., Kanazawa, A. M. & Green, A. E. (1994). *Tetrahedron Lett.* **35**, 105–108.

[bb3] Macrae, C. F., Bruno, I. J., Chisholm, J. A., Edgington, P. R., McCabe, P., Pidcock, E., Rodriguez-Monge, L., Taylor, R., van de Streek, J. & Wood, P. A. (2008). *J. Appl. Cryst.* **41**, 466–470.

[bb4] Montiel-Smith, S., Cervantes-Mejía, V., Dubois, J., Guénard, D., Guéritte, F. & Sandoval-Ramírez, J. (2002). *Eur. J. Org. Chem.* pp. 2260–2264.

[bb5] Rudolph, J., Sennhenn, P. C., Vlaar, C. P. & Sharpless, K. B. (1996). *Angew. Chem. Int. Ed.* **35**, 2810–2813.

[bb6] Sheldrick, G. M. (2008). *Acta Cryst.* A**64**, 112–122. 10.1107/S0108767307043930 18156677

[bb7] Siemens (1996). *XSCANS* Siemens Analytical X-ray Instruments Inc., Madison, Wisconsin, USA.

[bb8] Tinant, B., Declercq, J. P. & Cagnon, J. R. (1996). *Bull. Soc. Chim. Belg.* **105**, 325–328.

